# Clinical Evidence and FDA Recalls of Artificial Intelligence–Enabled Medical Devices

**DOI:** 10.1001/jamanetworkopen.2026.17920

**Published:** 2026-06-11

**Authors:** Yijun Ren, Yi Zheng, Daniel Windecker, Alan G. Fraser, George C. M. Siontis, Enrico G. Caiani

**Affiliations:** 1 Department of Electronics, Information and Bioengineering, Politecnico di Milano, Milan, Italy; 2 Department of Cardiology, Bern University Hospital, Inselspital, University of Bern, Bern, Switzerland; 3 Department of Diagnostic and Interventional Neuroradiology, University of Bern, Bern, Switzerland; 4 Department of Cardiology, University Hospital of Wales, Cardiff, the United Kingdom; 5 IRCCS Istituto Auxologico Italiano, Ospedale S. Luca, Milan, Italy

## Abstract

**Importance:**

Artificial Intelligence (AI)–enabled medical devices present new safety challenges related to algorithmic complexity and evolving performance. Identifying device characteristics that influence recalls would be essential to caution against premature, widespread clinical deployment and to suggest increased surveillance.

**Objective:**

To test the hypothesis that AI-enabled medical devices lacking sufficient clinical evidence at approval have a higher hazard of recall.

**Design, Setting, and Participants:**

This retrospective cohort study included AI-enabled medical devices authorized by the US Food and Drug Administration (FDA) between August 11, 1995, and August 31, 2024. Devices were assessed from FDA authorization to recall or administrative censoring up to August 31, 2024. Adverse event reports were obtained from the FDA Manufacturer and User Facility Device Experience database and by using the Coordinating Research and Evidence for Medical Devices Postmarket Surveillance (CORE-MD PMS) Tool. Reports were mapped to International Medical Device Regulators Forum (IMDRF) codes to summarize device-related issues.

**Exposure:**

Device characteristics and occurrence of device problems.

**Main Outcomes and Measures:**

The primary outcome was time from FDA authorization to recall. Time to recall was analyzed using a bayesian Weibull survival model with regularized horseshoe priors. Associations were quantified using hazard ratios (HRs) and 95% credible intervals (CrIs) derived from the bayesian model.

**Results:**

Among 903 AI-enabled medical devices, 43 (4.8%) were recalled after a median (IQR) interval of 458 (263-1092) days. Devices with missing information regarding clinical studies had a higher hazard of recall than devices with published clinical studies (HR, 1.39; 95% CrI, 0.84-3.52). Devices flagged in the CORE-MD PMS Tool (HR, 4.28; 95% CrI, 1.01-13.10) or in both databases (HR, 2.77; 95% CrI, 0.87-14.28) had higher recall hazards. IMDRF codes showed that problems related to temperature (occurred for 1 of 9 recalls; HR, 0.45; 95% CrI, 0.03-1.26) and labeling (occurred for 2 of 9 recalls; HR, 0.55; 95% CrI, 0.06-1.37) were associated with lower hazards of recall. Incorrect use of the AI-enabled device (12 of 31 devices) was also associated with a higher estimated hazard of recall (HR, 3.33; 95% CrI, 0.97-10.71).

**Conclusions and Relevance:**

In this study of 903 FDA-authorized AI-enabled medical devices, missing information on supporting clinical studies was associated with a higher chance of recall; devices with use-related problems also were associated with elevated recall hazards. These findings highlight the importance of robust clinical validation and strengthened postmarket oversight for AI-enabled devices.

## Key Points

**Question:**

Which artificial intelligence (AI)–enabled medical device characteristics, such as availability of clinical performance studies and the nature of postmarket issues, are associated with risk of recall?

**Findings:**

This cohort study of 903 medical devices found that devices with information missing from supporting clinical studies had an increased likelihood of recall compared with devices supported by such evidence. Use-related deviations most often triggered recalls, highlighting unique safety challenges of AI systems.

**Meaning:**

These findings suggest that device features and postmarket evidence jointly determine safety profiles of such devices and that establishing a unified surveillance framework could enhance safety signal detection.

## Teaser

This cohort study examines characteristics of artificial intelligence (AI)–enabled medical devices, including clinical study evidence, associated with US Food and Drug Administration (FDA) recall.

## Introduction

Artificial intelligence (AI)–enabled medical devices offer new possibilities across clinical practice,^1,2,3^ but their adoption has raised concerns regarding transparency and robustness of clinical evidence supporting their use.^4,5,6,7,8,9^ In fact, many received regulatory authorization without clinical validation.^10,11^ A recent cross-sectional evaluation of US Food and Drug Administration (FDA)–approved, AI-enabled medical devices found that clinical performance studies had been reported publicly for only one-half of devices, and those studies often lacked sufficient information to assess generalizability.^4^

AI-enabled medical devices without publicly available evidence may be more likely to be withdrawn from the market due to insufficient validation before approval. A study from 2021 found that for FDA-approved high-risk medical devices, most recalls involved devices approved via the 510(k) pathway.^12^ If it is confirmed that devices approved through faster or less rigorous pathways may be more prone to postmarket issues, it would support withholding wide clinical adoption of new AI-enabled medical devices until robust evidence is available, in favor of controlled market introduction with enhanced monitoring.

Adverse event reports submitted to the FDA Manufacturer and User Facility Device Experience (MAUDE) database constitute a key source for postmarket surveillance, including reports from mandatory (eg, manufacturers) and voluntary (eg, health care professionals) reporters. Additionally, field safety notices (FSNs) are communications from manufacturers about corrective actions,^13^ published on the websites of national regulatory authorities and mandatory under the current European Medical Device Regulation. FSNs may address issues ranging from minor concerns (eg, labeling errors) to more serious safety issues (eg, algorithmic malfunctions). Together, they provide complementary postmarket evidence, describing diverse device-level problems.^14,15^

A prior study^16^ identified associations of regulatory submission characteristics with recall risk using a linear probability model, while another study^17^ found higher recall frequency among devices without reported validation using logistic regression. However, the influence of postmarket safety problems on device recall remains unclear, and no study has yet applied bayesian approaches in this context.^18,19^

Accordingly, we tested the hypothesis that AI-enabled medical devices with limited or absent supporting clinical evidence at the time of their approval would have a higher hazard of recall. To do so, we examined (1) whether the availability of clinical performance studies is associated with time to recall, (2) which postmarket safety problems have the largest magnitude of association with recalls, and (3) whether bayesian methods can help to characterize factors associated with device recalls.

## Methods

### Study Design and Data Sources

This retrospective cohort study included all AI-enabled medical devices approved by the FDA between November 8, 1995, and August 31, 2024. Postmarket reports published up to August 31, 2024, were extracted from the FDA MAUDE database and by using the Coordinating Research and Evidence for Medical Devices Postmarket Surveillance (CORE-MD PMS) Tool.^20,21^ The reporting and performance of the study adheres to the Strengthening the Reporting of Observational Studies in Epidemiology (STROBE) reporting guideline.^22^ This study analyzed publicly available postmarket medical device report data and did not involve intervention, interaction, or identifiable private information from individuals. Accordingly, it did not constitute human-participants research under the US Common Rule, 45 CFR 46.102(e); informed consent and institutional review board approval were not required.

### Definition and Characteristics of AI-Enabled Medical Devices

The present analysis was performed on a previously defined sample of AI-enabled medical devices that had received FDA approval for clinical use and been listed on the FDA website^23^ from the date of the register’s inception until August 31, 2024.^4,24^ These devices comprise a broad and heterogeneous group of devices that incorporate or consist of AI-software, including implantable devices, hardware-based systems, imaging and diagnostic platforms, as well as software-only medical devices. No additional selection criteria were applied. All information was extracted from online sources, and summary reports were downloaded from the FDA website.^23^ We collected detailed descriptive information about each device, including any performance metrics and reported clinical evaluations, and also considered the nonavailability of these details as a variable for analysis.

### Adverse Events Reported on the FDA MAUDE Database

The MAUDE database provides postmarket safety data from the US, representing the largest medical device market. Because MAUDE contains reports submitted by users of medical devices,^25^ relevant files were downloaded and merged to create a unified database containing all reports published up to August 31, 2024.^26^ Records were retrieved using the names of manufacturers and of their devices and classified using FDA problem codes and International Medical Device Regulators Forum (IMDRF) codes, in particular Annex A for medical device problems.^27^ The 27 categories in the top level (ranging from A01 to A27) were considered to capture the main device problems, including category A11, specifically referring to software-related issues (written programs or AI systems) (see eTable 1 in Supplement 1). This process was enabled by the public availability of mapping between these codes as described in eAppendix 1 in Supplement 1. Binary variables (A01 [MAUDE] to A27 [MAUDE]) were created for each IMDRF code to indicate whether the problem had been reported for each device.

### Retrieval and Analysis of FSNs

The CORE-MD PMS web-scraping tool^20,21^ was used to extract and generate a structured database (CORE-DB) including all publicly available FSNs from the regulators’ websites of 18 countries: Croatia, Czechia, Denmark, Estonia, France, Germany, Greece, Ireland, Italy, Latvia, Netherlands, Poland, Portugal, Slovenia, Spain, Sweden, Switzerland, and the UK. The term FSN refers to the collective safety information available across these official websites.

We focused on FSNs published before August 31, 2024. Manufacturer and device names for all the AI-enabled devices were used to query the CORE-DB and retrieve any relevant FSNs. Duplicated FSNs were identified and removed; more details are given in eAppendix 2 in Supplement 1. Unique FSNs were then coded according to IMDRF codes (eTable 1 in Supplement 1). The classification was conducted independently by 2 investigators (Y.R. and Y.Z.), and possible discrepancies were resolved through discussion.

Binary variables (A01 [COREDB] to A27 [COREDB]) were created for each IMDRF code to indicate whether the problem had been reported for each device. These variables were then combined with another variable (Axx [MAUDE]*) *to construct a unified set of binary variables (A01 to A27), coded as 1 if either source equaled 1, or coded as 0 otherwise. To mitigate potential sparsity, only variables for which both categories (either absence or presence) had more than 5 observations were included in the analysis. Additionally, a categorical variable (postmarket database) was introduced to indicate the source of the signal: both (if both sources reported the device), CORE-DB, MAUDE, and none.

### Statistical Analysis

The unit of analysis was the individual AI-enabled medical device. Descriptive statistics were used to summarize device characteristics and frequencies of recall. The primary outcome was time from FDA authorization to recall, with devices censored at the study end date (August 31, 2024) if no recall occurred. Device characteristics, together with binary variables (Axx) indicating the occurrence of the corresponding device problems, were treated as independent exposures.

Associations with time to recall were evaluated using a bayesian Weibull proportional hazards model with regularized horseshoe priors to account for sparse events and multiple covariates. Let *t_i≥0_* denote the observed follow-up time for device *i*, with *i* = 1,…,*n,* and let *x_i_* denote the corresponding vector of *D* exposures. The hazard function for device *i *at time *t* was specified as:
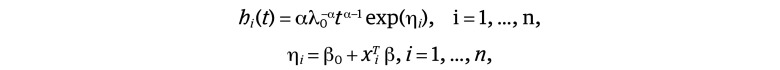
where α > 0 is the Weibull shape parameter, λ_0_ > 0 is the baseline scale parameter, β_0_ is the intercept, and β = (β,…β*_D_*) represents the vector of *D* regression coefficients. The cumulative incidence function for device *i*, representing the probability of recall by time *t,* was calculated with the following equation:

*F_i_*(*t*) = 1 − exp(−λ_0_^−α^t^α^ × exp(η*_i_*)).

To complete the specification of the bayesian model, weakly informative normal priors were assumed, calculated as:


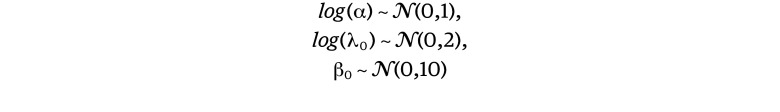
.

For the regression coefficients β, given the expected sparsity, the regularized horseshoe prior, a global-local shrinkage prior that keeps signals while shrinking noise, was employed.^28,29^ The prior is defined hierarchically as:
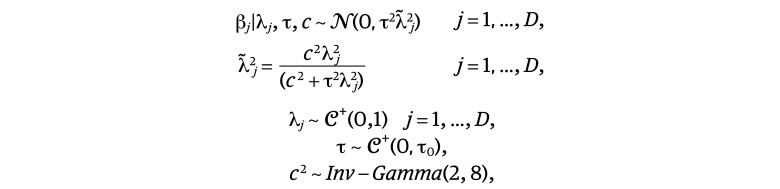
where λ*_j_* indicates local shrinkage parameters, τ is a global shrinkage parameter, and *c* is a slab scale parameter that limits the magnitude of large coefficients. The choice of the prior for *c* reflects a weak informative prior. The global scale τ_0_ was defined based on prior guess *p*_0_ for the expected number of relevant exposures as^28^: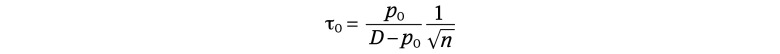.To determine a suitable value for *p*_0_*_,_* the goodness-of-fit indexes were evaluated, such as the widely applicable information criterion^30^ and log-pseudo marginal likelihood,^31^ for *p*_0_ = 5, *p*_0_ = 10, and *p*_0_ = 15. We observed only minimal differences in model performance across different values of *p*_0_. Therefore, the more parsimonious model (*p*_0_ = 5) was chosen for all subsequent analyses.

Statistical analysis was conducted between May 2025 and March 2026. The analysis was performed using Stan via the R interface *rstan *(R version 4.3.3 [R Project for Statistical Computing).^32^ The model was run in Stan for 55 000 iterations, with the first 5000 discarded as burn-in. Applying a thinning interval of 10 yielded 5000 posterior samples. Posterior regression coefficients were exponentiated to obtain posterior distributions of hazard ratios (HR). Point estimates were summarized using the posterior medians, and 95% credible intervals (CrI) were defined as the 2.5th and 97.5th percentiles of the exponentiated posterior samples. Evidence of a protective effect was defined as at least an 80% posterior probability that the HR was below 1 (ie, at least 80% of the exponentiated posterior samples were less than 1), and increased risk was defined as at least an 80% posterior probability that the HR was above 1 (ie, at least 80% of the exponentiated posterior samples were greater than 1).

## Results

**Figure 1.**
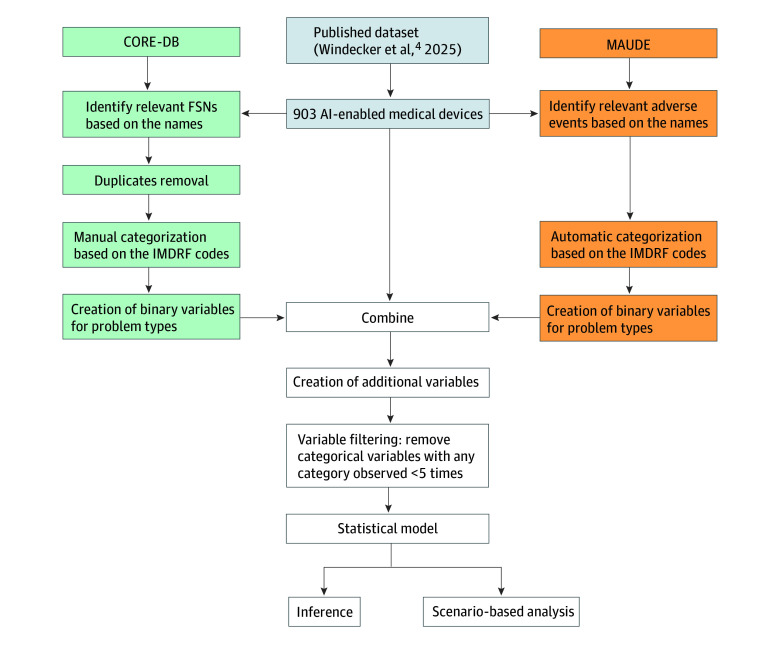
Flowchart Summarizing the Design of This Study Datasets used in this study included the Coordinating Research and Evidence Database (CORE-DB) from the CORE for Medical Devices (CORE-MD) Postmarket Surveillance Tool; a published dataset from Windecker et al,^4^ obtained via the US Food and Drug Administration (FDA) website; and the FDA Manufacturer and User Facility Device Experience (MAUDE) database. AI indicates artificial intelligence; FSN, field safety notice; IMDRF, International Medical Device Regulators Forum.

For the total of 903 devices, 179 unique FSNs were identified concerning 39 devices (4.3%), while 43 (4.8%) of the 903 AI-enabled devices had been recalled.^4^ The median (IQR) time from approval to recall was 458 (263-1092) days (approximately 15 [9-36] months). An overview of the process of combining datasets and merging data for this study is provided in Figure 1.

### Analysis of FSNs From the CORE-DB

Of 909 matched FSNs published before August 31, 2024, 686 were considered relevant after removing those that did not pertain to the device itself (eg, related only to accessories). After removal of duplicate reports, there were 179 unique FSNs (eFigure in Supplement 2). The agreement between independent raters, assessed using the Cohen κ, was 0.80, confirming the consistency of FSN classification according to the IMDRF codes.

### Exploratory Analysis

Table 1 reports the characteristics of FDA-authorized AI-enabled medical devices. Higher recall rates were observed for implanted AI-enabled devices (6 implanted; 2 recalled [33.3%]), for devices without clinical performance studies (218 devices; 17 recalled [7.8%]), for those classified as software-device combinations (239 devices; 29 recalled [12.1%]), and for devices flagged by both MAUDE and CORE-DB (16 devices; 8 recalled [50.0%]). These patterns may suggest possible risk factors; however, they should be interpreted with caution due to the small sample size. Table 2 summarizes the binary variable (Axx), presenting for each code the number and percentage of recalled and not recalled devices, restricted to those with more than 5 observations in both categories (20 of 27 codes). The problems with AI-enabled devices that had been reported most frequently were related to usage or failure to employ the software as expected by the manufacturer (IMDRF code A23; cited for 12 recalled devices, corresponding to 1.3% of 903 devices), issues with operation of the software (IMDRF code A11; cited for 11 devices, corresponding to 1.2% of 903 devices), and concerns about the output from the device or deviation of its results from what was expected (A09; cited for 10 devices, corresponding to 1.1% of 903 devices).

**Table 1.** Characteristics of FDA-Authorized, AI-Enabled Medical Devices

Variable	Description	Category	Count	Recalled No. (%)
Clinical performance study	Studies conducted to evaluate the safety, effectiveness, and real-world performance of the AI device in a clinical setting	No	218	17 (7.8)
		Yes	505	13 (2.6)
		Missing	180	13 (7.2)
Development study	Research conducted during the design and training phase of the AI model to develop and validate its algorithms	No	812	42 (5.2)
		Yes	91	1 (1.1)
DICOM adherence	Conformance with the DICOM standard for handling, storing, transmitting, and displaying medical imaging data	Yes	559	20 (3.6)
		Missing	344	23 (6.7)
Eligible for third-party review	Indicates whether the device qualifies for review by an FDA-accredited third party instead of direct FDA review	Eligible	437	24 (5.5)
		Ineligible	466	19 (4.1)
FDA decision summary	An official FDA document outlining the regulatory decision, supporting evidence, and rationale for device clearance or approval	No	857	39 (4.6)
		Yes	46	4 (8.7)
Geographic origin of the applicant	The origin of the applicant reported in the submitted documents	Asia	154	7 (4.6)
		Europe	183	8 (4.4)
		North America	467	25 (5.4)
		Other	99	3 (3.0)
Implanted device	Indicates whether the device is designed to be placed inside the body	No	897	41 (4.6)
		Yes	6	2 (33.3)
Panel	The medical field or clinical discipline the device is intended for	Cardiovascular	91	3 (3.3)
		Hematology	17	3 (17.7)
		Neurology	29	1 (3.5)
		Radiology	692	30 (4.3)
		Other^a^	74	6 (8.1)
Physical state	Standalone software as a medical device, or software in a medical device	Standalone software	664	14 (2.1)
		Software and device	239	29 (12.1)
Postmarket database	Source of the postmarket adverse events	Both	16	8 (50.0)
		CORE-DB	25	7 (28.0)
		MAUDE	36	6 (16.7)
		None	826	22 (2.7)
Summary malfunction reporting	A regulatory reporting mechanism allowing manufacturers to submit grouped summaries of certain device malfunctions rather than individual reports	Eligible	552	37 (6.7)
		Ineligible	351	6 (1.7)

Abbreviations: AI, artificial intelligence; CORE-DB, Coordinating Research and Evidence for Medical Devices Database; FDA, US Food and Drug Administration; MAUDE, Manufacturer and User Facility Device Experience.

^a^ Other refers to clinical chemistry, dental, ear-nose-throat, general and plastic surgery, general hospital, immunology, microbiology, obstetrics and gynecology, orthopedic, pathology, and physical medicine.

**Table 2.** Summary of Device Counts and Recall Rates for IMDRF Categories, Using Data From MAUDE and CORE-DB

IMDRF code and case	Absence of device problem			Presence of device problem		
	Count	Recalled, No. (%)	SD, %	Count	Not recalled, No. %	SD, %
A01: Patient device interaction	896	41 (4.6)	0.7	7	2 (28.6)	17.1
A02: Manufacturing and packaging	892	40 (4.5)	0.7	11	3 (27.3)	13.4
A04: Material integrity	891	41 (4.6)	0.7	12	2 (16.7)	10.8
A05: Mechanical	885	39 (4.4)	0.7	18	4 (22.2)	9.8
A07: Electrical	892	40 (4.5)	0.7	11	3 (27.3)	13.4
A09: Output	877	33 (3.8)	0.6	26	10 (38.5)	9.5
A10: Temperature	893	42 (4.7)	0.7	10	1 (10.0)	9.5
A11: Computer software	865	32 (3.7)	0.6	38	11 (28.9)	7.4
A12: Connection	896	42 (4.7)	0.7	7	1 (14.3)	13.2
A13: Communication	893	38 (4.3)	0.7	10	5 (50.0)	15.8
A15: Activation, positioning	897	41 (4.6)	0.7	6	2 (33.3)	19.2
A16: Protective measures	897	41 (4.6)	0.7	6	2 (33.3)	19.2
A17: Compatibility	895	40 (4.5)	0.7	8	3 (37.5)	17.1
A18: Contamination	896	42 (4.7)	0.7	7	1 (14.3)	13.2
A21: Labeling	894	41 (4.6)	0.7	9	2 (22.2)	13.9
A22: Human-device interface	896	39 (4.4)	0.7	7	4 (57.1)	18.7
A23: Usage	872	31 (3.6)	0.6	31	12 (38.7)	8.7
A24: No identified device or problem	887	38 (4.3)	0.7	16	5 (31.2)	11.6
A25: No apparent adverse event	894	39 (4.4)	0.7	9	4 (44.4)	16.6
A26: Insufficient information	885	34 (3.8)	0.6	18	9 (50.0)	11.8

Abbreviations: CORE-DB, Coordinating Research and Evidence for Medical Devices Database; IMDRF, International Medical Device Regulators Forum; MAUDE, Manufacturer and User Facility Device Experience.

### Posterior Inference and Parameter Interpretation

**Figure 2.**
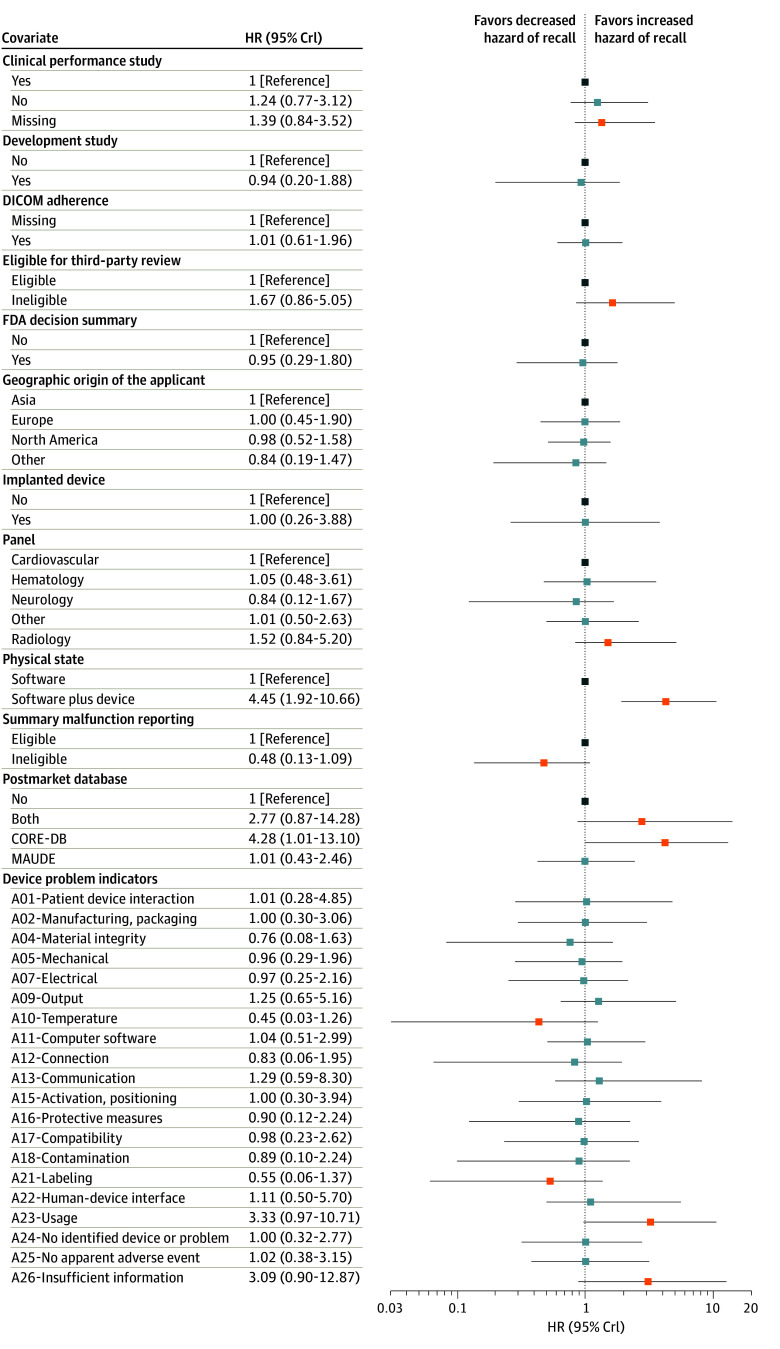
Descriptive Forest Plot of Hazard of Recall Points represent posterior medians of hazard ratios (HRs), and horizontal lines indicate 95% credible intervals (CrIs); covariates with significant outcomes are highlighted in orange, while nonsignificant outcomes are highlighted in light blue. For categorical variables, HRs are interpreted relative to the specified reference category (dark blue dots). HRs greater than 1 indicate an increased hazard of recall, whereas hazard ratios less than 1 indicate a decreased hazard. CORE-DB indicates Coordinating Research and Evidence for Medical Devices Database; DICOM, digital imaging and communications in medicine standard; FDA, US Food and Drug Administration; MAUDE, Manufacturer and User Facility Device Experience.

Figure 2 presents the posterior HR with 95% CrI for each covariate. Several characteristics were associated with a higher hazard of recall. Software-device combinations showed the largest magnitude of association (HR, 4.45; 95% CrI, 1.92-10.66). Devices flagged by CORE-DB alone (HR, 4.28; 95% CrI, 1.01-13.10) or by both databases (2.77; 95% CrI, 0.87-14.28) also exhibited elevated hazards of recall. Ineligibility for third-party review (HR, 1.67; 95% CrI, 0.86-5.05), radiology panel (HR, 1.52; 95% CrI, 0.84-5.20), and missing information regarding clinical performance study (HR, 1.39; 95% CrI, 0.84-3.52) were likewise associated with higher hazard of recall. In contrast, ineligibility for summary malfunction reporting was associated with a lower hazard of recall (HR, 0.48; 95% CrI, 0.13-1.09). Regarding the reported software-device problems, issues related to A10 (temperature; HR, 0.45; 95% CrI, 0.03-1.26) and A21 (labeling; HR, 0.55; 95% CrI, 0.06-1.37) were associated with a lower hazard of recall (but they had been reported for only 1 and 2 recalled devices, respectively), while A23 (usage; HR, 3.33; 95% CrI, 0.97-10.71) and A26 (insufficient information; HR, 3.09; 95% CrI, 0.90-12.87) problems were associated with a higher hazard of recall.

### Scenario-Based Analysis of Estimated Cumulative Recall Risk

To provide a practical interpretation of the results, the cumulative probability of recall for a baseline device was computed. It was defined to represent a typical scenario in our dataset: a nonimplantable radiology, AI-enabled medical device submitted by an applicant based in North America, developed as a combined software-device system, compliant with Digital Imaging and Communications in Medicine requirements, without any FDA decision summary, ineligible for third-party review, without development studies, and eligible for summary malfunction reporting.

Table 3 reports the estimated cumulative incidence of recall for the baseline profile at different time points, stratified by covariates, clinical performance study and postmarket database, and the types of problems identified as significant exposures (Figure 2). When no safety signals were identified in either databases, the cumulative recall risk remained low with only a small, expected increase over time. With the absence of a clinical performance study, the probability rose from 0.42% (95% CrI, 0.10%-1.67%) at 30 days to 4.91% (95% CrI, 1.63%-16.81%) at 365 days, and 5.40% (95% CrI, 1.62%-19.16%)when information was missing, compared with 3.69% (95% CrI, 1.15%-12.67%)with a clinical performance study present. Marked differences emerged across problem type and signal source. The highest risks were observed for usage problems (A23), particularly when reported in both databases or in CORE-DB alone; when A23 was reported by CORE-DB and no clinical study was conducted, the cumulative recall probability increased from 5.34% (95% CrI, 0.77%-29.32%) at 30 days to 48.80% (95% CrI, 9.83%-98.26%) at 365 days, reaching 52.64% (95% CrI, 10.38%-99.27%) in case of missing clinical study information. The wide credible intervals, particularly at 365 days, reflect substantial uncertainty, likely due to limited information and increased extrapolation at longer follow-up times. In contrast, the lowest risk was observed for temperature problems (A10) reported only in MAUDE in presence of clinical studies, increasing modestly from 0.14% (95% CrI, 0.01%-1.06%) to 1.69% (95% CrI, 0.08%-10.35%) over 365 days. These findings suggest that clinical evidence could mitigate recall risk, even within high-impact problem categories.

**Table 3.** Estimated Cumulative Incidence of Recall for the Baseline Device Profile^a^

IMDRF code	Estimated cumulative probability of recall, median % (95% CrI)											
	Clinical performance study: present				Clinical performance study: absent				Clinical performance study: missing			
	30 d	90 d	180 d	365 d	30 d	90 d	180 d	365 d	30 d	90 d	180 d	365 d
None	0.31 (0.07-1.33)	0.93 (0.24,-3.56)	1.84 (0.53-6.76)	3.69 (1.15-12.67)	0.42 (0.10-1.67)	1.23 (0.34-4.49)	2.47 (0.75-8.62)	4.91 (1.63-16.81)	0.46 (0.10-1.99)	1.36 (0.34-5.42)	2.70 (0.74-10.14)	5.40 (1.62-19.16)
A10: temperature problem												
Both	0.34 (0.02-3.38)	1.02 (0.06-9.30)	2.06 (0.13-17.31)	4.13 (0.28-31.82)	0.46 (0.03-4.28)	1.36 (0.09-11.52)	2.73 (0.19-21.39)	5.47 (0.40-39.33)	0.50 (0.03-5.03)	1.51 (0.10-14.20)	3.02 (0.21-25.67)	6.02 (0.44-44.72)
CORE-DB	0.53 (0.02-4.14]	1.58 (0.08-10.79)	3.14 (0.16-20.24)	6.26 (0.33-36.78)	0.70 (0.03-5.39)	2.12 (0.10-14.67)	4.23 (0.20-26.68)	8.39 (0.40-46.53)	0.78 (0.04-6.64)	2.32 (0.12-17.40)	4.60 (0.23-30.84)	9.00 (0.48-54.09)
MAUDE	0.14 (0.01-1.06)	0.42 (0.02-2.86)	0.84 (0.04-5.41)	1.69 (0.08-10.35)	0.18 (0.01-1.36)	0.56 (0.02-3.71)	1.12 (0.05-7.20)	2.26 (0.11-13.66)	0.20 (0.01-1.61)	0.62 (0.03-4.46)	1.24 (0.06-8.33)	2.51 (0.13-15.97)
A21: labeling												
Both	0.41 (0.0-5.39)	1.22 (0.08-14.53)	2.39 (0.16-25.90)	4.80 (0.34-43.93)	0.55 (0.03-6.38)	1.63 (0.10-17.42)	3.22 (0.22-31.40)	6.46 (0.46-53.74)	0.59 (0.04-7.34)	1.75 (0.12-19.48)	3.51 (0.26-35.11)	7.03 (0.54-57.83)
CORE-DB	0.62 (0.04-4.55)	1.88 (0.14-12.01)	3.71 (0.29-21.59)	7.41 (0.64-38.26)	0.81 (0.06-5.92)	2.46 (0.19-15.77)	4.88 (0.39-28.21)	9.68 (0.83-48.73)	0.89 (0.06-6.87)	2.68 (0.21-17.40)	5.33 (0.44-30.91)	10.60 (0.94-52.40)
MAUDE	0.16 (0.01-1.23)	0.49 (0.04-3.29)	0.98 (0.07-6.07)	1.98 (0.16-11.64)	0.21 (0.01-1.55)	0.64 (0.05-4.24)	1.30 (0.10-8.20)	2.61 (0.21-15.18)	0.23 (0.02-1.77)	0.70 (0.06-4.93)	1.39 (0.12-9.12)	2.80 (0.25-17.06)
A23: usage												
Both	2.81 (0.31-22.99)	8.13 (1.00-52.00)	15.57 (2.02-76.13)	29.10 (4.12-94.76)	3.71 (0.43-27.76)	10.76 (1.42-60.33)	20.32 (2.88-84.43])	37.10 (5.79-97.74)	4.07 (0.43-32.25)	11.91 (1.38-66.53)	22.27 (2.77-88.82)	40.02 (5.65-98.88)
CORE-DB	4.01 (0.58-22.30)	11.49 (1.83-50.75)	21.95 (3.70-76.20)	39.38 (7.19-94.64)	5.34 (0.77-29.32)	15.20 (2.50-62.82)	28.13 (5.05-86.28)	48.80 (9.83-98.26)	5.89 (0.83-33.44)	16.78 (2.65-69.11)	30.84 (5.34-90.37)	52.64 (10.38-99.27)
MAUDE	1.06 (0.15-6.43)	3.18 (0.51-17.01)	6.24 (1.07-30.27)	12.15 (2.22-51.89)	1.39 (0.20-8.43)	4.14 (0.67-22.38)	8.11 (1.40-39.80)	15.71 (2.87-63.73)	1.55 (0.22-9.74)	4.59 (0.74-25.15)	8.91 (1.53-43.06)	17.34 (3.02-67.80)
A26: insufficient information												
Both	2.56 (0.27-25.63)	7.49 (0.86-59.04)	14.56 (1.71-82.74)	27.62 (3.30-97.49)	3.42 (0.39-31.08)	9.99 (1.19-65.66)	18.92 (2.38-87.62)	34.75 (4.63-98.81)	3.80 (0.36-37.90)	11.07 (1.17-76.59)	20.98 (2.27-94.90)	38.06 (4.34-99.79)
CORE-DB	3.84 (0.36-31.68)	11.03 (1.12-67.12)	20.81 (2.21-88.42)	37.76 (4.33-98.77)	5.06 (0.49-40.64)	14.69 (1.43-78.22)	27.34 (2.82-95.51)	47.50 (5.57-99.84)	5.52 (0.49-49.46)	15.73 (1.41-85.85)	28.99 (2.82-98.15)	50.49 (5.56-99.97)
MAUDE	0.96 (0.13-7.44)	2.85 (0.43-19.87)	5.63 (0.89-35.05)	11.04 (1.81-57.66)	1.28 (0.18-9.34)	3.84 (0.57-24.79)	7.54 (1.17-43.22)	14.82 (2.39-68.06)	1.41 (0.17-12.10)	4.20 (0.54-31.32)	8.35 (1.12-52.62)	16.20 (2.30-78.02)

Abbreviations: CORE-DB, Coordinating Research and Evidence for Medical Devices Database; IMDRF, International Medical Device Regulators Forum; MAUDE, Manufacturer and User Facility Device Experience.

^a^ Estimates are presented across categories of the covariates (clinical performance study and postmarket database) and the type of problem identified by the reporting sources according to IMDRF codes.

## Discussion

In this cohort study, we identified few approved AI-enabled medical devices that were formally withdrawn from the market. However, our findings show how their characteristics, regulatory attributes, and postmarket evidence could influence the risk of voluntary recall.

### Importance of Clinical Performance Studies

The model identified missing information on clinical performance studies as one of the most significant factors associated with an increase in recall risk for AI-enabled medical devices. Robust and representative clinical validation is important to ensure the effectiveness and safety of AI-enabled medical devices^17^ because insufficient or unrepresentative validation may introduce bias and limit real-world generalizability.^33,34^ Our findings suggest that the current premarket evaluation framework may not fully capture real-world clinical variability. Although this limitation is not unique to AI-enabled devices, their data-driven and context-dependent nature may amplify the consequences of inadequate validation, underscoring the need for more rigorous and transparent clinical performance evaluation.

### Association of Problem Types With Recall Risk

To our knowledge, no previous study has systematically explored the association of device problems categorized according to IMDRF codes with the risk of recall. Although these codes apply broadly to medical devices, the relative importance of specific problems may vary by the type of device. For example, battery- and software-related malfunctions predominate in implantable pacemakers and defibrillators,^35^ while manufacturing- and packaging-related problems are more common for total knee implants.^14,15^ In contrast, our analysis of AI-enabled medical devices identified use-related issues and insufficient information as being associated with a higher risk of recall. Use-related problems may arise when devices are deployed in situations that differ from the original settings; without robust evidence of generalizability, both safety and effectiveness may be compromised.^4^ Insufficient information may conceal high-risk malfunctions that were not accurately described and addressed, while targeted postmarket monitoring could detect safety signals earlier.

Notably, safety problems identified through CORE-DB, alone or also in MAUDE, were associated with a higher hazard of recall. This finding may reflect differences in reporting mechanisms. MAUDE contains adverse event reports from different end-users, whereas CORE-DB captures manufacturer-issued FSNs, highlighting the value of integrating multiple postmarket data sources.

### Bayesian Modeling Framework for Safety Signal Detection

Previous work analyzed the odds of any recall using multivariate logistic regression,^17^ whereas we applied a bayesian time-to-event framework to model time to recall. Specifically, the regularized horseshoe prior^28^ was adopted to enable automatic variable selection and continuous shrinkage, allowing the model to identify the most important factors from more than 30 candidate variables while preventing overfitting. Despite the limited dataset size, this uncertainty-aware approach ensures robust inference and reliable estimation of recall risk over time, highlighting the value of bayesian models for postmarket assessment of AI-enabled medical devices.

### Role of Regulatory Oversight in Recall Risk

The model identified that several variables related to FDA regulatory procedures were associated with the hazard of recall. Devices ineligible for third-party review showed a higher hazard of recall, which could be attributed to higher inherent risk; devices with high-risk profile or those requiring a De Novo submission are excluded from third-party review and must be submitted directly to the FDA for evaluation.^36^ Also, devices ineligible for summary malfunction reporting were associated with a lower hazard of recall, likely reflecting stronger evidence requirements. Enhanced regulatory oversight and more controlled testing conditions could reduce performance issues and thus recalls of devices in real-world use.

### Limitations

This study has several limitations. First, to identify approved AI-enabled devices we used exclusively the list available from the FDA website, so the study may reflect characteristics specific to the US regulatory environment. The model could be applied to datasets from other regulatory authorities if similar information becomes available. Second, interactions between exposures were not analyzed because the limited data size, together with the sparsity of the exposures, constrained the feasibility of modeling higher-order effects. Third, the AI-enabled medical devices that were analyzed encompass a broad spectrum of software algorithms. While we accounted for differences across medical specialties, other factors such as data input type, intended function or risk classes, could also influence recall risk, and would warrant further investigation.

## Conclusions

In this retrospective cohort study of 903 FDA-authorized AI-enabled medical devices, missing publicly available information on supporting clinical studies was associated with a higher hazard of recall. Postmarket data showed that recalls were more frequently associated with usage-related problems than with temperature or labeling issues, highlighting unique safety challenges of AI systems. Overall, device features and postmarket evidence jointly determine safety profiles. A unified surveillance framework could enhance early detection of safety signals, and future work should include multijurisdictional analyses and time-to-recall modeling to capture evolving safety risks.

## Notes

Supplement 1. eAppendix 1. Datasets Used eAppendix 2. FSN Retrieval eTable 1. Description of the IMDRF Level 1 Terms eTable 2. Manual Assignment of FDA Problem Codes Lacking the Corresponding IMDRF Codes eFigure. Flowchart of Selection Process of Field Safety Notices Relevant to the AI-Enabled Medical Devices Included in the Analysis

Supplement 2. Data Sharing Statement

## References

[1] Topol EJ. Toward the eradication of medical diagnostic errors. Science. 2024;383(6681):eadn9602. doi:10.1126/science.adn9602 38271508

[2] Abernethy A, Adams L, Barrett M, . The promise of digital health: then, now, and the future. NAM Perspect. 2022;2022. doi:10.31478/202206e 36177208 PMC9499383

[3] Acosta JN, Falcone GJ, Rajpurkar P, Topol EJ. Multimodal biomedical AI. Nat Med. 2022;28(9):1773-1784. doi:10.1038/s41591-022-01981-2 36109635

[4] Windecker D, Baj G, Shiri I, . Generalizability of FDA-approved AI-enabled medical devices for clinical use. JAMA Netw Open. 2025;8(4):e258052. doi:10.1001/jamanetworkopen.2025.8052 40305017 PMC12044510

[5] Fraser AG, Biasin E, Bijnens B, . Artificial intelligence in medical device software and high-risk medical devices—a review of definitions, expert recommendations and regulatory initiatives. Expert Rev Med Devices. 2023;20(6):467-491. doi:10.1080/17434440.2023.2184685 37157833

[6] Warraich HJ, Tazbaz T, Califf RM. FDA perspective on the regulation of artificial intelligence in health care and biomedicine. JAMA. 2025;333(3):241-247. doi:10.1001/jama.2024.21451 39405330

[7] Siontis GCM, Sweda R, Noseworthy PA, Friedman PA, Siontis KC, Patel CJ. Development and validation pathways of artificial intelligence tools evaluated in randomised clinical trials. BMJ Health Care Inform. 2021;28(1):e100466. doi:10.1136/bmjhci-2021-100466 34969668 PMC8718483

[8] Muralidharan V, Adewale BA, Huang CJ, . A scoping review of reporting gaps in FDA-approved AI medical devices. NPJ Digit Med. 2024;7(1):273. doi:10.1038/s41746-024-01270-x 39362934 PMC11450195

[9] Han R, Acosta JN, Shakeri Z, Ioannidis JPA, Topol EJ, Rajpurkar P. Randomised controlled trials evaluating artificial intelligence in clinical practice: a scoping review. Lancet Digit Health. 2024;6(5):e367-e373. doi:10.1016/S2589-7500(24)00047-5 38670745 PMC11068159

[10] Chouffani El Fassi S, Abdullah A, Fang Y, . Not all AI health tools with regulatory authorization are clinically validated. Nat Med. 2024;30(10):2718-2720. doi:10.1038/s41591-024-03203-3 39187696

[11] Jain SS, Goto S, Hall JL, . Pragmatic approaches to the evaluation and monitoring of artificial intelligence in health care: a science advisory from the American Heart Association. Circulation. Published online November10, 2025. doi:10.1161/CIR.0000000000001400 41208719

[12] Dubin JR, Simon SD, Norrell K, Perera J, Gowen J, Cil A. Risk of recall among medical devices undergoing us food and drug administration 510(k) clearance and premarket approval, 2008-2017. JAMA Netw Open. 2021;4(5):e217274. doi:10.1001/jamanetworkopen.2021.7274 33956132 PMC8103223

[13] European Union EUR-Lex. Regulation (EU) 2017/745 of the European Parliament and of the Council of 5 April 2017 on medical devices, amending directive 2001/83/EC, regulation (EC) No 178/2002 and regulation (EC) No 1223/2009 and repealing council directives 90/385/EEC and 93/42/EEC (text with EEA relevance). Published March 20, 2023. Accessed January 28, 2024. https://data.europa.eu/eli/reg/2017/745/2023-03-20/eng

[14] Hoogervorst LA, Ren Y, Melvin T, . Safety notices and registry outlier data measure different aspects of safety and performance of total knee implants: a comparative study of safety notices and register outliers. Acta Orthop. 2024;95:667-676. doi:10.2340/17453674.2024.42361 39584822 PMC11587162

[15] Ren Y, Hoogervorst LA, Caiani EG, . Frequency of safety signals from scientific reports, manufactures, registers, and other sources for a random selection of hip and knee prostheses. Acta Orthop. 2025;96:460-466. doi:10.2340/17453674.2025.44035 40557841 PMC12188684

[16] Everhart AO, Sen S, Stern AD, Zhu Y, Karaca-Mandic P. Association between regulatory submission characteristics and recalls of medical devices receiving 510(k) clearance. JAMA. 2023;329(2):144-156. doi:10.1001/jama.2022.22974 36625811 PMC9857565

[17] Lee B, Kramer P, Sandri S, . Early recalls and clinical validation gaps in artificial intelligence–enabled medical devices. JAMA Health Forum. 2025;6(8):e253172. doi:10.1001/jamahealthforum.2025.3172 40844774 PMC12374217

[18] Gelman A, Carlin JB, Stern HS, Dunson DB, Vehtari A, Rubin DB. Bayesian Data Analysis. Chapman and Hall/CRC; 1995. doi:10.1201/9780429258411

[19] Hespanhol L, Vallio CS, Costa LM, Saragiotto BT. Understanding and interpreting confidence and credible intervals around effect estimates. Braz J Phys Ther. 2019;23(4):290-301. doi:10.1016/j.bjpt.2018.12.006 30638956 PMC6630113

[20] Ren Y, Bertoldi M, Fraser AG, Caiani EG. Validation of CORE-MD PMS support tool: a novel strategy for aggregating information from notices of failures to support medical devices’ post-market surveillance. Ther Innov Regul Sci. 2023;57(3):589-602. doi:10.1007/s43441-022-00493-y 36652105 PMC10133046

[21] Ren Y, Caiani EG. Leveraging natural language processing to aggregate field safety notices of medical devices across the EU. NPJ Digit Med. 2024;7(1):352. doi:10.1038/s41746-024-01337-9 39632973 PMC11618595

[22] von Elm E, Altman DG, Egger M, Pocock SJ, Gøtzsche PC, Vandenbroucke JP; STROBE Initiative. The Strengthening the Reporting of Observational Studies in Epidemiology (STROBE) statement: guidelines for reporting observational studies. Lancet. 2007;370(9596):1453-1457. doi:10.1016/S0140-6736(07)61602-X 18064739

[23] U.S. Food & Drug Administration. Artificial intelligence-enabled medical devices. Updated March 4, 2026. Accessed May 5, 2026. https://www.fda.gov/medical-devices/software-medical-device-samd/artificial-intelligence-and-machine-learning-aiml-enabled-medical-devices

[24] AI in cardiovascular medicine FDA devices. GitHub. Accessed May 6, 2026. https://github.com/AI-in-Cardiovascular-Medicine/FDA-devices

[25] Liebel TC, Daugherty T, Kirsch A, Omar SA, Feuerstein T. *Analysis*: Using the FDA MAUDE and Medical Device Recall Databases to Design Better Devices. Biomed Instrum Technol. 2020;54(3):178-188. doi:10.2345/0899-8205-54.3.178 32442013

[26] Ensign LG, Cohen KB. A primer to the structure, content and linkage of the FDA’s manufacturer and user facility device experience (MAUDE) files. EGEMS (Wash DC). 2017;5(1):12. doi:10.5334/egems.221 29930960 PMC5994953

[27] IMDRF Adverse Event Terminology Working Group. IMDRF terminologies for categorized adverse event reporting (AER): terms, terminology structure and codes. Published March 18, 2020. Accessed May 5, 2026. https://www.imdrf.org/sites/default/files/docs/imdrf/final/technical/imdrf-tech-200318-ae-terminologies-n43.pdf

[28] Piironen J, Vehtari A. Sparsity information and regularization in the horseshoe and other shrinkage priors. Electron J Stat. 2017;11(2):5018-5051. doi:10.1214/17-EJS1337SI

[29] Piironen J, Vehtari A. On the hyperprior choice for the global shrinkage parameter in the horseshoe prior. Proceedings of Machine Learning Research. Published 2017. Accessed August 6, 2025. https://proceedings.mlr.press/v54/piironen17a.html

[30] Watanabe S. A widely applicable Bayesian information criterion. *J Mach Learn Res*. 2013;14:867-897.

[31] Chen MH, Huang L, Ibrahim JG, Kim S. Bayesian variable selection and computation for generalized linear models with conjugate priors. Bayesian Anal. 2008;3(3):585-614. doi:10.1214/08-BA323 19436774 PMC2680310

[32] Stan Development Team. RStan: the R interface to stan. Accessed July 15, 2025. https://mc-stan.org/rstan/

[33] Mittermaier M, Raza MM, Kvedar JC. Bias in AI-based models for medical applications: challenges and mitigation strategies. NPJ Digit Med. 2023;6(1):113. doi:10.1038/s41746-023-00858-z 37311802 PMC10264403

[34] Celi LA, Cellini J, Charpignon ML, . for MIT Critical Data. Sources of bias in artificial intelligence that perpetuate healthcare disparities-A global review. PLOS Digit Health. 2022;1(3):e0000022. doi:10.1371/journal.pdig.0000022 36812532 PMC9931338

[35] Ren Y, Baruffini A, Biolo S, . Clinical significance of field safety notices concerning implantable pacemakers and defibrillators. Europace. 2026;28(2):euag031. doi:10.1093/europace/euag031 41731706 PMC12962233

[36] Miller BJ, Blanks W, Yagi B. The 510(k) third party review program: promise and potential. J Med Syst. 2023;47(1):93. doi:10.1007/s10916-023-01986-5 37642768 PMC10465388

