# Supplementary material for: Clinical Evidence and FDA Recalls of Artificial Intelligence–Enabled Medical Devices
Source: JAMA Netw Open. 2026 Jun 11;9(6):e2617920. doi: 10.1001/jamanetworkopen.2026.17920 (PMC13261491; doi:10.1001/jamanetworkopen.2026.17920)
Supplement: Supplement 1. — eAppendix 1. Datasets Used eAppendix 2. FSN Retrieval eTable 1. Description of the IMDRF Level 1 Terms eTable 2. Manual Assignment of FDA Problem Codes Lacking the Corresponding IMDRF Codes eFigure. Flowchart of Selection Process of Field Safety Notices Relevant to the AI-Enabled Medical Devices Included in the Analysis [file jamanetwopen-e2617920-s001.pdf]

Supplemental Online Content

Ren Y, Zheng Y, Windecker D, Fraser AG, Siontis GCM, Caiani EG. Clinical evidence and FDA recalls of artificial intelligence–enabled medical devices. *JAMA Netw. Open*. 2026;9(6):e2617920. doi:10.1001/jamanetworkopen.2026.17920

eAppendix 1. Datasets Used

eAppendix 2. FSN Retrieval

eTable 1. Description of the IMDRF Level 1 Terms

eTable 2. Manual Assignment of FDA Problem Codes Lacking the Corresponding IMDRF Codes

eFigure. Flowchart of Selection Process of Field Safety Notices Relevant to the AI-Enabled Medical Devices Included in the Analysis

This supplemental material has been provided by the authors to give readers additional information about their work.

© 2026 Ren Y et al. *JAMA Network Open*.

eAppendix 1. Datasets Used

Three separate datasets, downloaded from the official FDA websites, were used: the dataset of devices with reported problems, the dataset of event records, and the dataset of reported problems coded according to the FDA problem codes. These sources were linked through a unique key, "MDR_REPORT_KEY". The first step was to retrieve all relevant "MDR_REPORT_KEY" values from the device database by matching manufacturer and device names. Once these keys were identified, they were used to extract the corresponding event records from the record dataset. Then, using the reported-problems dataset, each event was associated with the specific FDA problem code. As each "MDR_REPORT_KEY" identified in the first step was also linked back to the specific device in our list of interest, we could generate for each device the list of reported problems in FDA codes. Finally, using the publicly available mapping between FDA codes and IMDRF codes, each device was associated with the corresponding IMDRF codes reported by the MAUDE database. It should be noted that 22 FDA codes did not have a direct IMDRF equivalent. For these cases, we assigned the most appropriate IMDRF Level 1 code manually, as detailed in **eTable 2**.

This mapping enabled structured analysis of the device problems and allowed harmonization of problem types across different regulatory sources, as IMDRF provides an internationally recognized vocabulary for adverse event classification.

eAppendix 2. FSN Retrieval

To identify relevant FSNs from the CORE-DB, a two-stage process was applied. First, manufacturer-based filtering was applied, identifying all FSNs associated with the corresponding manufacturer. Subsequently, device name matching was conducted to further refine the results by identifying those FSNs in which the device name encompassed the target term, irrespective of word order. More specifically, both the manufacturer and device names were converted to lowercase and tokenized into individual words. Then, regular expressions with word-boundary constraints were used to verify the matches. This method enhances the precision of the resulting dataset while ensuring the retained FSNs were pertinent to the analysis.

After retrieving the FSNs of interest from the CORE-DB, a post-processing step was performed to remove all notices published after the device recall date (i.e., only for those had been recalled). For each FSN, the following information was retrieved: *Manufacturer, Device, Date*, and *URL* (i.e., the link to the original website where the FSN was officially published). From the URL, the corresponding PDF file was also downloaded for further processing.

Since manufacturers are required to notify the national authority in each country where their devices are put on the market about any encountered issues, different national authorities or regulatory agencies may publish the same FSN, leading to duplicated FSNs for the same problem and device. To address this, duplicated FSNs were identified as those issued by the same manufacturer for the same underlying device problem, and only the first one in chronological order was retained. Afterwards, the resulting set of unique FSNs was manually categorised according to the IMDRF coding.

© 2026 Ren Y et al. *JAMA Network Open*.

eTable 1. Description of the IMDRF Level 1 Terms

IMDRF code	IMDRF description of medical device problem
A01 – Patient Device Interaction Problem	Problem related to the interaction between the patient and the device.
A02 – Manufacturing, Packaging or Shipping Problem	Problem associated with any deviations from the documented specifications of the device that relate to nonconformity during manufacture to the design of an item or to specified manufacturing, packaging or shipping processes (out of box problem).
A03 – Chemical Problem	Problem associated with any from the documented specifications of the device that relate to any chemical characterization, i.e. element, compound, or mixture.
A04 – Material Integrity Problem	Problem associated with any deviations from the documented specifications of the device that relate to the limited durability of all material used to construct device.
A05 – Mechanical Problem	Problems associated with mechanical actions or defects, including moving parts or subassemblies, etc.
A06 – Optical Problem	Problem associated with transmission of visible light affecting the quality of the image transmitted or otherwise affecting the intended application of the visible light path.
A07 – Electrical /Electronic Property Problem	Problem associated with the function of the electrical circuitry of the device.
A08 – Calibration Problem	Problem associated with the operation of the device, related to its accuracy, and associated with the calibration of the device.
A09 – Output Problem	Problem associated with any deviation from the documented specifications of the device that relate to the end result, data, or test results provided by the device.
A10 – Temperature Problem	Problem associated with the device producing unintended temperatures.
A11 – Computer Software Problem	Problem associated with written programs, codes, and/or software system that affects device performance or communication with another device.
A12 – Connection Problem	Problem associated with linking of the device and/or the functional units set up to provide means for a transfer of liquid, gas, electricity or data.
A13 – Communication or Transmission Problem	Problem associated with the device sending or receiving signals or data. This includes transmission among internal components of the device to which the device is intended to communicate.
A14 – Infusion or Flow Problem	Problem associated with the device failing to deliver or draw liquids or gases as intended (e.g. delivering drugs at incorrect rate, problems with drawing fluid from a system). This includes vacuum collection devices and manual or mechanical pumps.

© 2026 Ren Y et al. *JAMA Network Open*.

IMDRF code	IMDRF description of medical device problem
A15 – Activation, Positioning or Separation Problem	Problem associated with any deviations from the documented specifications of the device that relate to the sequence of events for activation, positioning or separation of device. Note: Deployment is synonymous with activation.
A16 – Protective Measures Problem	Problem associated with any deviations from the documented specifications of the device that relate to the implemented and inherited design features specific to devices used for reducing risks to patient or caregiver or maintaining risks within specified levels.
A17 – Compatibility Problem	Problem associated with compatibility between device, patients or substances (medication, body fluid, etc.)
A18 – Contamination /Decontamination Problem	Problem associated with the presence of any unexpected foreign substance found in the device, on its surface or in the package materials, which may affect performance or intended use of the device, or problem that compromise effective decontamination of the device.
A19 – Environmental Compatibility Problem	Problem associated with the surrounding conditions in which the device is being used such as temperature, noise, lighting, ventilation, or other external factors such as power supply.
A20 – Installation-Related Problem	Problem associated with unsatisfactory installation, configuration, and/or setup of a specific device.
A21 – Labelling, Instructions for Use or Training Problem	Problem associated with device markings/labelling, instructions for use, training and maintenance documentation or guidelines.
A22 – Human-Device Interface Problem	Problem associated with an act or omission of an act that has a different result than that intended by the manufacturer or expected by the operator.
A23 – Use of Device Problem	Problem associated with failure to process, service, or operate the device according to the manufacturer's recommendations or recognized best practices.
A24 – Adverse Event Without Identified Device or Use Problem	An adverse event (e.g. patient harm) appears to have occurred, but there does not appear to have been a problem with the device or the way it was used.
A25 – No Apparent Adverse Event	A report has been received but the description provided does not appear to relate to an adverse event. This code allows a report to be recorded for administration purposes, even if it doesn't meet the requirements for adverse event reporting.
A26 – Insufficient Information	An adverse event appears to have occurred but there is not yet enough information available to classify the device problem.
A27 – Appropriate Term/Code Not Available	The device problem is not adequately described by any other term. Note: this code must not be used unless there is no other feasible code. The preferred term should be documented when submitting an adverse event report. This information will be used to determine if a new term should be added to the code table.

© 2026 Ren Y et al. *JAMA Network Open*.

*e*Table 2. Manual Assignment of FDA Problem Codes Lacking the Corresponding IMDRF Codes

FDA term	IMDRF Level 1 code
Bent	A04 – Material Integrity Problem
Charred	A10 – Temperature Problem
Detachment Of Device Component	A05 – Mechanical Problem
Component Falling	A26 – Insufficient Information
Application Interface Becomes Non-Functional Or Program Exits Abnormally	A11 – Computer Software Problem
Device Expiration Issue	A26 – Insufficient Information
Image Reversal	A09 – Output Problem
Difficult To Position	A15 – Activation, Positioning or Separation Problem
Device Stops Intermittently	A26 – Insufficient Information
Inadequate Storage	A19 – Environmental Compatibility Problem
Device Inoperable	A26 – Insufficient Information
Invalid Sensing	A07 – Electrical /Electronic Property Problem
Device Issue	A26 – Insufficient Information
Incorrect Or Inadequate Test Results	A09 – Output Problem
Battery Impedance Issue	A07 – Electrical /Electronic Property Problem
Device Operates Differently Than Expected	A26 – Insufficient Information
Device Operational Issue	A26 – Insufficient Information
Electronic Property Issue	A07 – Electrical /Electronic Property Problem
Human Factors Issue	A22 – Human-Device Interface Problem
Improper Device Output	A09 – Output Problem
Physical Property Issue	A26 – Insufficient Information
Programming Issue	A11 – Computer Software Problem

© 2026 Ren Y et al. *JAMA Network Open*.

eFigure. Flowchart of Selection Process of Field Safety Notices Relevant to the AI-Enabled Medical Devices Included in the Analysis

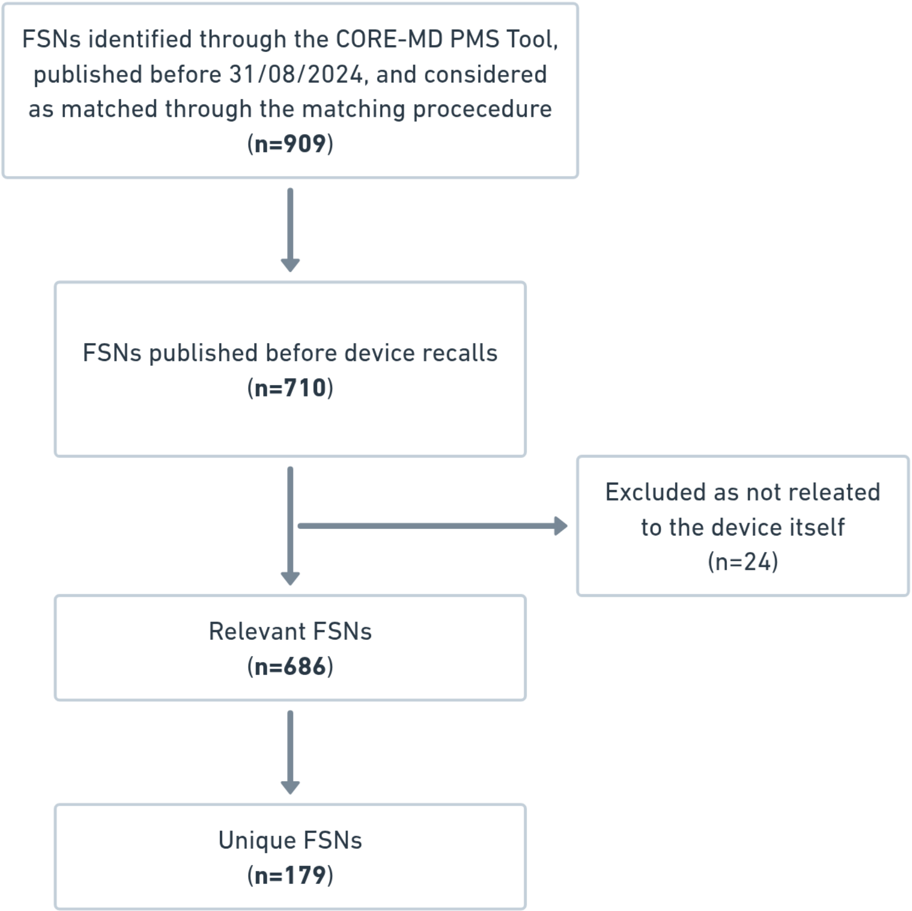

© 2026 Ren Y et al. *JAMA Network Open*.
