# Supplementary material for: Clinical Evidence and FDA Recalls of Artificial Intelligence–Enabled Medical Devices
Source: JAMA Netw Open. 2026 Jun 11;9(6):e2617920. doi: 10.1001/jamanetworkopen.2026.17920 (PMC13261491; doi:10.1001/jamanetworkopen.2026.17920)
Supplement: Supplement 2. — Data Sharing Statement [file jamanetwopen-e2617920-s002.pdf]

Data Sharing Statement

Ren. Clinical Evidence and FDA Recalls of Artificial Intelligence–Enabled Medical Devices. *JAMA Netw Open*. Published June 11, 2026. doi:10.1001/jamanetworkopen.2026.17920

Data
Data available: Yes
Data types: Data (not involving human participants)
How to access data: https://github.com/yijun-r/prediction-fda-ai-md-recalls
When available: With publication

Supporting Documents
Document types: None

Additional Information
Who can access the data: Researchers whose proposed use of the data has been approved.
Types of analyses: The data will be made available for research purpose.
Mechanisms of data availability: The data will be made available upon request, with investigator support as needed.
